# Analysis of risk factors of low cardiac output syndrome after congenital heart disease operation: what can we do

**DOI:** 10.1186/s13019-021-01518-7

**Published:** 2021-05-17

**Authors:** Bangrong Song, Haiming Dang, Ran Dong

**Affiliations:** Department of Cardiac Surgery, Beijing Anzhen Hospital, Capital Medical University, No.2 Anzhen Road, Chaoyang, Beijing, 100029 China

**Keywords:** Low cardiac output syndrome, Congenital heart disease, Cardiology, Pediatric, Surgery, Treatment, Care

## Abstract

**Background:**

It’s necessary to analyze the related risk factors and complications of low cardiac output syndrome (LCOS) after operation in children with congenital heart disease (CHD), to elucidate the management strategy of LCOS.

**Methods:**

CHD children admitted to the department of cardiology in our hospital from January 15, 2019 to October 31, 2020 were included. The personal and clinical data of CHD children with LCOS and without LCOS were collected and compared. Logistic regression analyses were conducted to identify the risk factors of postoperative LCOS. Besides, the complication and mortality of LCOS and no LCOS patients were compared.

**Results:**

A total of 283 CHD patients were included, the incidence of postoperative LCOS in CHD patients was 12.37%. There were significant differences in the age, preoperative oxygen saturation, two-way ventricular shunt, duration of CPB and postoperative residual shunt between two groups (all *p* < 0.05). Logistic regression analyses indicated that age ≤ 4y(OR2.426, 95%CI1.044 ~ 4.149), preoperative oxygen saturation ≤ 93%(OR2.175, 95%CI1.182 ~ 5.033), two-way ventricular shunt (OR3.994, 95%CI1.247 ~ 6.797), duration of CPB ≥ 60 min(OR2.172, 95%CI1.002 ~ 4.309), postoperative residual shunt (OR1.487, 95%CI1.093 ~ 2.383) were the independent risk factors of LCOS in patients with CHD (all *p* < 0.05). There were significant differences in the acute liver injury, acute kidney injury, pulmonary infection, tracheotomy, duration of mechanical ventilation, length of ICU stay and mortality (all *p* < 0.05), no significant difference in the 24 h drainage was found(*p* = 0.095).

**Conclusion:**

LCOS after CHD is common, more attentions should be paid to those patients with age ≤ 4y, preoperative oxygen saturation ≤ 93%, two-way ventricular shunt, duration of CPB ≥ 60 min, postoperative residual shunt to improve the prognosis of CHD patients.

## Background

Congenital heart disease (CHD) is kind of heart disease caused by abnormal development of the heart and blood vessels in the fetus, and it is very common in the clinical pediatric cardiology [1]. Because of its inability to heal itself, relevant surgery treatment is needed for patients with CHD [2]. The early diagnosis and treatment of CHD is essential to the prognosis of patients. Correction of congenital malformations by cardiopulmonary bypass (CPB) under direct vision is the main surgical method for the treatment of CHD [3]. Low cardiac output syndrome (LCOS) is a common serious complication after the operation of CHD, the incidence of LOCS after operation varies from 9.08 to 21.25%, and it is also the major cause of death of children after operation [4, 5]. Therefore, the prevention of postoperative LCOS in CHD patients is of great significance to improve the prognosis of patients.

In recent years, with the improvement of medical materials and surgical techniques, the incidence of postoperative LCOS has been reduced [6, 7]. However, the occurrence of postoperative LCOS is related to higher mortality and poor prognosis [8]. Therefore, reducing the incidence of postoperative LCOS in children with CHD is important to the prognosis of CHD patients. At present, there are few studies on the risk factors of LCOS in children after CHD. In this present study, we aimed to analyze the clinical data of children with CHD, to identify the risk factors of LCOS in children after CHD and their influence on the clinical prognosis of CHD patients, thereby providing reliable evidences to the clinical LCOS preventions and CHD managements.

## Methods

### Ethics approval

In this study, all methods were performed in comply with the relevant guidelines and regulations. Our study had been approved by the ethics committee of our hospital (XC201811029a), and written informed consents had been obtained from the relatives or guardians of the included CHD children.

### Patients

We selected children with CHD who were admitted to the department of cardiology in our hospital from January 15, 2019 to October 31, 2020 as the research population. The inclusion criteria of this study were: ①The diagnosis of CHD in all children was confirmed by cardiac color Doppler ultrasound. ②All children underwent intracardiac malformation correction surgery under CPB in our hospital. ③The family members of the patients were informed and agreed to participate in this study. The exclusion criteria of this study were: ①children who underwent emergency surgery; ②age > 14 years old; ③children who received cardiac interventional therapy; ④Patients with missing clinical data.

### The diagnosis of LCOS

The diagnosis of LCOS was made if patients met more than two of following diagnostic criteria [9, 10]: ① Heart index < 2 L·min^− 1^·m^− 2^; ② Systolic blood pressure < 90 mmHg or systolic blood pressure decreased by more than 20% compared with preoperative blood pressure; ③Central venous pressure > 15cmH_2_O; ④The difference between the central temperature and the peripheral temperature > 5 °C, and the limbs were cold; ⑤Urine volume < 0.5 ml·kg^− 1^·h^− 1^ for more than 2 h. According to whether LCOS occurred after operation, the patients were divided into LCOS group and no LCOS group.

### Data collections

Two authors collected and recorded the preoperative, intraoperative and postoperative clinical data with unified form, including the diagnosis of CHD, gender, age(y), preoperative oxygen saturation(%), inner diameter of left atrium (mm), end diastolic diameter of left ventricle (mm), end systolic diameter of left ventricular (mm), left ventricular ejection fraction (%), inner diameter of pulmonary artery (mm), atrial and ventricular shunt, preoperative laboratory examination (white blood cell count, platelet count, red blood cell count, creatinine, alanine aminotransferase, aspartate aminotransferase), duration of surgery (min),duration of CPB (min), infusion of red blood cell suspension and postoperative residual shunt. The related variables of the two groups of children’s risk factors were statistically compared, and risk factors were screened.

### Statistical processing

All data in this study are statistically analyzed using SPSS 23.0 software. Measurement data were expressed as mean ± standard deviation, and comparisons between groups were conducted by t test. Count data were expressed as the number of cases and percentages, and comparisons between groups were conducted by chi-square test. We performed multivariate logistic regression analysis on variables with significant statistical significance in univariate analysis between groups to explore the risk factors that affected children with LCOS after CHD surgery. In this study, the difference was statistically significant if *P* < 0.05.

## Results

### Patients

A total of 283 CHD patients were included, of whom 35 patients had LCOS after surgery, the incidence of postoperative LCOS in CHD patients was 12.37%. The type distributions of CHD were presented in Table 1. As indicated in Table 2, there were significant differences in the age, preoperative oxygen saturation, two-way ventricular shunt, duration of CPB and postoperative residual shunt (all *p* < 0.05), and there were no significant differences in the gender, inner diameter of left atrium, end diastolic diameter of left ventricle, end systolic diameter of left ventricular, left ventricular ejection fraction, inner diameter of pulmonary artery, atrial shunt, white blood cell count, platelet count, red blood cell count, creatinine, alanine aminotransferase, aspartate aminotransferase, duration of surgery and infusion of red blood cell suspension (all *p* > 0.05).

**Table 1:** The types distribution of CHD

Type of CHD	LCOS group(*n* = 35)	No LCOS group(*n* = 248)	χ^2^	*P*
Ventricular septal defect	14 (40%)	103(%)	1.297	0.084
Atrial septal defect	8 (22.85%)	52 (20.97%)	1.102	0.087
Pulmonary artery stenosis	4 (11.43%)	29 (11.69%)	1.177	0.104
Tetralogy of Fallot	7 (20%)	44 (17.74%)	1.015	0.118
Right ventricular double outlet	1 (2.86%)	11 (4.44%)	1.272	0.123
Pulmonary vein ectopic drainage	1 (2.86%)	9 (3.63%)	1.136	0.096

**Table 2:** The characteristics of included patients

Variables	LCOS group(*n* = 35)	No LCOS group(*n* = 248)	t/χ^2^	*P*
Male/female	19/16	136/112	1.089	0.071
Age(y)	3.12 ± 2.58	6.91 ± 5.24	1.124	0.002
Preoperative oxygen saturation(%)	91.62 ± 10.31	94.23 ± 10.55	7.033	0.041
Inner diameter of left atrium (mm)	17.93 ± 7.18	17.17 ± 8.41	3.126	0.085
End diastolic diameter of left ventricle (mm)	26.25 ± 8.24	27.57 ± 7.09	7.104	0.098
End systolic diameter of left ventricular (mm)	17.43 ± 7.11	17.52 ± 7.14	4.116	0.102
Left ventricular ejection fraction (%)	67.41 ± 8.33	68.12 ± 7.51	10.274	0.117
Inner diameter of pulmonary artery (mm)	21.28 ± 9.26	20.86 ± 9.33	4.201	0.079
Atrial shunt				
Left to right shunt	13 (37.15%)	93 (37.50%)	1.192	0.085
No shunt	18 (51.43%)	121 (48.79%)	1.169	0.059
Right to left shunt	2 (5.71%)	19 (7.66%)	1.202	0.076
Two-way shunt	2 (5.71%)	15 (6.05%)	1.113	0.069
Ventricular shunt				
Left to right shunt	12 (34.29%)	95 (38.31%)	1.120	0.072
No shunt	17 (48.57%)	124 (50%)	1.286	0.066
Right to left shunt	2 (5.71%)	19 (7.66%)	1.113	0.084
Two-way shunt	4 (11.43%)	10 (4.03%)	1.208	0.038
Preoperative laboratory examination				
White blood cell count (× 10^9^ ·L^−1^)	9.03 ± 1.61	8.93 ± 1.23	1.128	0.077
Platelet count (×10^9^ ·L^−1^)	213.71 ± 84.52	210.24 ± 123.12	1.246	0.104
Red blood cell count(×10^9^ ·L^−1^)	4.73 ± 1.17	4.66 ± 1.14	1.093	0.071
Creatinine (μmol·L^−1^)	34.13 ± 14.36	33.26 ± 15.23	2.146	0.085
Alanine aminotransferase (U ·L^−1^)	18.27 ± 10.12	17.13 ± 12.56	3.108	0.063
Aspartate aminotransferase(U ·L^−1^)	19.34 ± 12.32	19.46 ± 15.30	2.997	0.102
Duration of surgery (min)	125.52 ± 66.49	124.59 ± 75.17	12.084	0.105
Duration of CPB (min)	67.73 ± 22.97	51.09 ± 23.95	9.169	0.013
Infusion of red blood cell suspension (mL)	224.36 ± 98.14	228.65 ± 92.17	18.104	0.098
Postoperative residual shunt	4 (11.43%)	3 (1.21%)	1.297	0.018

### Logistic regression analysis

The variable assignments of multivariate logistic regression were presented in Table 3. As Table 4 showed, logistic regression results indicated that age ≤ 4y(OR2.426, 95%CI1.044 ~ 4.149), preoperative oxygen saturation ≤ 93%(OR2.175, 95%CI1.182 ~ 5.033), two-way ventricular shunt (OR3.994, 95%CI1.247 ~ 6.797), duration of CPB ≥ 60 min(OR2.172, 95%CI1.002 ~ 4.309), postoperative residual shunt (OR1.487, 95%CI1.093 ~ 2.383) were the independent risk factors of LCOS in patients with CHD (all *p* < 0.05).

**Table 3:** The variable assignment of multivariate logistic regression

Factors	Variables	Assignment
LCOS	Y	Yes = 1, no = 2
Age(y)	X_1_	≤4 = 1, > 4 = 2
Preoperative oxygen saturation(%)	X_2_	≤93 = 1, > 93 = 2
Two-way ventricular shunt	X_3_	Yes = 1, no = 2
Duration of CPB (min)	X_4_	≥60 = 1, < 60 = 2
Postoperative residual shunt	X_5_	Yes = 1, no = 2

**Table 4:** Logistic regression analysis on the risk factors of LCOS in patients with CHD

Variables	β	S^−^x	OR	95%CI	*P*
Age ≤ 4y	0.103	0.217	2.426	1.044 ~ 4.149	0.012
Preoperative oxygen saturation ≤ 93%	0.127	0.220	2.175	1.182 ~ 5.033	0.025
Two-way ventricular shunt	0.131	0.127	3.994	1.247 ~ 6.797	0.006
Duration of CPB ≥ 60 min	0.146	0.170	2.172	1.002 ~ 4.309	0.043
Postoperative residual shunt	0.109	0.113	1.487	1.093 ~ 2.383	0.027

### The postoperative complications and prognosis comparison

As presented in Table 5, there were significant differences in the acute liver injury, acute kidney injury, pulmonary infection, tracheotomy, duration of mechanical ventilation, length of ICU stay and mortality (all *p* < 0.05), no significant difference in the 24 h drainage was found(*p* = 0.095).

**Table 5:** The postoperative complications and prognosis of patients with CHD

Variables	LCOS group(*n* = 35)	No LCOS group(*n* = 248)	t/χ^2^	*P*
24 h drainage (mL)	136.63 ± 53.98	128.04 ± 52.04	22.107	0.095
Acute liver injury	3 (8.57%)	7 (2.82%)	1.214	0.019
Acute kidney injury	6 (17.14%)	9 (3.63%)	1.082	0.006
Pulmonary infection	3 (8.57%)	6 (1.72%)	1.079	0.012
Tracheotomy	5 (14.29%)	2 (0.81%)	1.128	0.001
Duration of mechanical ventilation(h)	32.13 ± 23.28	10.28 ± 7.84	7.202	0.027
Duration of ICU stay(d)	8.10 ± 3.15	3.07 ± 2.55	2.409	0.018
Mortality	4 (11.43%)	2 (0.81%)	1.128	0.001

## Discussions

LCOS is one of the common complications after heart surgery, and it is also an important cause of death in children after CHD [11]. Studies have reported that the incidence of LCOS after heart surgery can be as high as 25.16%. Although studies [12, 13] have reported that the occurrence of LCOS may be related to preoperative cardiac function, intraoperative operations and CPB, there are still no exact indicators to reflect the risk of its occurrence. Therefore, exploring the risk factors of LCOS is of great significance to the prevention and treatment of LCOS after CHD in children. At present, domestic and foreign studies on the risk factors of LCOS after CHD in children are inconsistent. It is believed that the occurrence of LCOS is caused by multiple factors, including impaired systolic and diastolic function of the heart, changes in cardiac load, and activation of inflammatory transmitters [14, 15]. The results of our study have found that age ≤ 4y, preoperative oxygen saturation ≤ 93%, two-way ventricular shunt, duration of CPB ≥ 60 min, postoperative residual shunt were the independent risk factors of LCOS in patients with CHD. Clinically, these risk factors should be identified early, and relevant intervention measures should be taken as soon as possible to reduce the occurrence of LCOS.

The younger the age, the higher the incidence of L COS, which may be associated to the incomplete development of myocardial cells in younger children and the susceptibility to ischemia and hypoxia [16, 17]. At the same time, most of the younger children who require early surgery have severe disease and deformity, LCOS is more likely to occur after surgery [18]. The incidence of LCOS in children with preoperative ventricular two-way shunt was significantly higher than that of non-shunt or other shunts [19]. Ventricular horizontal bidirectional shunt mostly occurs in the late stage of left-to-right shunt CHD such as ventricular septal defect with pulmonary hypertension [20]. The possible cause of postoperative LCOS may be increased pulmonary artery pressure before surgery or increased pulmonary circulation, which may lead to increased pulmonary vascular resistance in children after surgery [21]. Meanwhile, the systemic inflammatory response caused by CPB damages the pulmonary vascular endothelium and changes the vascular inflammatory response, increases the production of thromboxane, and reduces the production of endogenous NO, which causes pulmonary vasoconstriction and pulmonary vascular microthrombosis, leading to pulmonary blood vessels [22, 23]. A further increase in resistance increases the right ventricular afterload, which further leads to the occurrence of right heart failure and LCOS [24].

The lung ischemia-reperfusion injury caused by CPB also causes damage to the alveolar epithelial-endothelial barrier, leading to pulmonary congestion and pulmonary edema, and restricting oxygenation [25]. Meanwhile, CPB can lead to local postoperative ischemia-reperfusion injury [26]. The systemic inflammatory reaction increases the body’s energy requirements, makes the body in a high metabolic state, increases myocardial oxygen consumption, increases cardiac work, and further aggravates cardiac function damage [27]. Postoperative residual shunt is a common complication after CHD surgery in children, with an incidence of 5 to 25% [24, 28, 29]. The immediate postoperative residual shunt mainly occurs in children with intracardiac malformations with severe pulmonary hypertension [30]. At the time of unidirectional valve or artificial stoma, most of these children have severe disease and poor basic cardiac function [31]. The abnormal hemodynamics caused by residual shunt can aggravate the myocardial damage [32], and finally lead to LCOS.

The complications of LCOS after CPB operation can casuse adverse consequences for the patients. This study has found that children in the LCOS group have increased postoperative complications and increased mortality. The possible reasons may be related to following reasons: LCOS causes the insufficient perfusion of important organs such as liver, kidney, brain, leading to function damage [33]. The pulmonary circulatory congestion caused by LCOS causes pulmonary interstitial edema, affects the blood oxygen exchange of the lungs, and increases the incidence of pulmonary complications [34]. At present, there is a consensus that LCOS after open heart surgery is a risk factor for poor prognosis after cardiovascular disease [35]. Therefore, through strict monitoring of various indicators of cardiac output, early diagnosis of LCOS, and timely search for the cause and treatment, the effects of reducing complications and improving prognosis can be achieved [36]. Clinically, the timing of surgery should be strictly controlled according to the actual conditions of the child’s disease, age, etc. Before surgery, heart failure should be actively controlled and pulmonary artery pressure should be lowered [37]. During surgery, fine operations should be performed to reduce residual shunts and secondary operations. At the same time, surgical techniques should be continuously improved, surgical methods should be improved, and the aortic occlusion time should be shortened as much as possible [38]. Besides, it is necessary to improve the perioperative management and the recognition of LCOS, early detection and treatment are warranted.

This study has certain clinical significance for identifying the risk factors of postoperative LCOS in children with CHD and provides reference value for the prevention of LCOS. However, this study has certain limitations. This study is a single-center retrospective analysis with a small sample size, it is difficult to classify and compare the specific type of CHD. Therefore, it is necessary to design a rigorous multi-center prospective studies for better identifying the risk factors of LCOS, to provide insights into the clinical management of CHD.

## Conclusions

In conclusion, the incidence of LCOS after CHD is high and it’s closely associated with the prognosis of CHD patients. For CHD patients with age ≤ 4y, preoperative oxygen saturation ≤ 93%, two-way ventricular shunt, duration of CPB ≥ 60 min, postoperative residual shunt, they may have higher risk of LCOS. Those risk factors should be early identified and intervened to reduce the onset of LCOS. Limited by sample size, future studies with larger sample size in difference areas are warranted to further elucidate the risk factors of LCOS, to provide reliable evidences to the management of CHD.

## Data Availability

All data generated or analyzed during this study are included in this published article.

## Abbreviations

LCOS: Low cardiac output syndrome
CHD: Congenital heart disease
CPB: Cardiopulmonary bypass
